# Childhood-Onset COPA Syndrome Recognized Retrospectively in the Context of Polyarticular Juvenile Idiopathic Arthritis and Rheumatoid Arthritis

**DOI:** 10.1155/2023/3240245

**Published:** 2023-06-13

**Authors:** Roko P. A. Nikolic, Cristina Moran Toro

**Affiliations:** ^1^Department of Medicine, University of Calgary, Cumming School of Medicine, Calgary, Canada; ^2^Division of Rheumatology, Department of Medicine, University of Calgary, Cumming School of Medicine, Calgary, Canada

## Abstract

COPA syndrome is a very rare autoinflammatory disorder manifesting with childhood-onset arthritis and pulmonary and renal disease, of which awareness may remain lacking. We present the case of a twenty-year-old male patient seen in the Young Adults with Rheumatic Disease clinic. Initially diagnosed with seropositive polyarticular juvenile idiopathic arthritis, the patient's early childhood complaints of fatiguability, paroxysmal dyspnea, and pneumonia-like episodes were long to be felt unrelated to his arthritis. Upon transition to adult rheumatology care, a thorough review of the patient's history prompted imaging which revealed interstitial lung disease. Restrictive spirometry and genetic testing confirmed the retrospective diagnosis of COPA syndrome.

## 1. Introduction

COPA syndrome is a rare, monogenic autoinflammatory disorder characterized by arthritis and pulmonary and renal disease [1–3], the awareness of which may remain lacking amongst clinicians. Symptoms arise due to a mutation in coatomer subunit alpha (COPA), leading to malfunctional intracellular vesicular trafficking [1–3], and stimulator of interferon genes (STING) signaling [1]. COPA syndrome typically manifests in early childhood, and features may include pulmonary hemorrhage, exertional dyspnea and fatiguability, chronic cough, suspected recurrent respiratory tract infections, and polyarthritis [2–10]. Pulmonary disease ranges from interstitial lung disease to pulmonary hemorrhage, and patients often display restrictive spirometry, ground-glass opacification, and cystic changes [2, 3, 6, 7, 9]. Glomerular renal disease may be seen [2]. Polyarticular arthritis is another common feature of COPA syndrome [2]. Though knees, acral interphalangeal joints, ankles, and wrists are often involved [2–8, 10], involvement of the cervical spine [4], shoulders, and temporomandibular joints has also been reported [7]. Arthritis may be erosive [8]. COPA syndrome may be seronegative or variably seropositive for antinuclear antibody (ANA), antineutrophil cytoplasmic antibodies (ANCAs), rheumatoid factor (RF), and anticyclic citrullinated peptide (anti-CCP); if positive, ANCAs may be of the myeloperoxidase (MPO) or proteinase-3 (PR3) subtypes [2–10]. Extractable nuclear antigen seropositivity has also been reported [8].

## 2. Case Report

We present a twenty-year-old male patient seen in the Young Adults with Rheumatic Diseases (YARD) clinic. He was first diagnosed with seropositive polyarticular juvenile idiopathic arthritis (JIA) at the age of two when he presented with ankles, knee, and wrist arthritis. Prior to the JIA diagnosis, the patient experienced recurrent episodes of pneumonia and required multiple childhood hospital admissions for the same. While he did experience childhood fevers, these occurred in the context of pulmonary infections. No history of childhood rashes was available, and it is unknown whether interleukin levels were assessed in childhood. Initially managed with methotrexate in addition to ibuprofen and oral glucocorticoids, all treatments were discontinued after approximately 9 years of treatment as the patient was felt to be in remission.

**Figure 1:**
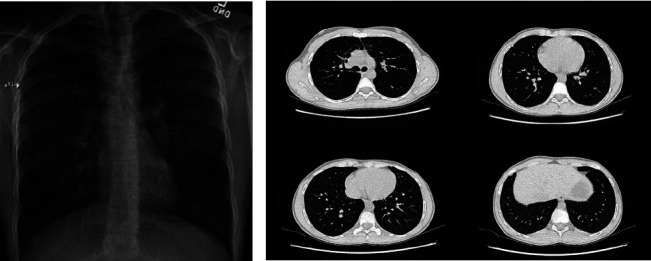
(a) Posterior-anterior chest X-ray reveling diffuse, fine reticulonodular haziness. (b) Lung-window computed tomography (CT) scan revealing small cystic spaces (0.2–0.4 millimeters) and ground-glass micronodularity.

The patient was reassessed by pediatric rheumatology at the age of fifteen, with a profile significant for short stature and recurrent bouts of pneumonia through childhood. He re-presented on account of intermittent elbow, shoulder, digital, and back arthralgias, morning stiffness lasting one hour, and oral sores. Physical examination revealed bilateral wrist effusions with limited extension, left knee flexion contracture, left subtalar and acral proximal interphalangeal joint restriction, and left first metacarpophalangeal joint effusion. No skin or nail changes were identified. Pertinent investigations included elevated immunoglobulin A with normal immunoglobulins G and M, strong RF (282 international units per milliliter) and anti-CCP (34.5 units per milliliter) seropositivity, normal C-reactive protein, elevated erythrocyte sedimentation rate (26 millimeters per hour), and negative human leukocytic antigen (HLA) B27. Urinalysis was unremarkable, and the patient's creatinine has remained within normal range. Magnetic resonance imaging revealed erosive wrist and ankle arthritis, subchondral cysts, bone marrow edema, and simple effusions. Ongoing disease activity indicated a switch from etanercept to abatacept. He was subsequently transferred to the YARD clinic. Functional capacity assessment revealed long-standing exercise intolerance and fatiguability since early childhood in comparison to peers and childhood-onset episodes of chest pressure, shortness of breath, and palpitations seemingly responsive to salbutamol. A chest X-ray revealed interstitial markings, and reticulonodular changes (Figure 1(a)). Spirometry demonstrated restrictive lung disease. A subsequent computed tomography scan demonstrated parenchymal lung disease with cystic changes and ground-glass nodularity (Figure 1(b)). Targeted genetic analysis confirmed the presence of a pathogenic mutation in the COPA gene. Further pathologic genetic findings were not reported. The patient was referred to pulmonology, who confirmed the diagnosis. The patient remains in the care of pulmonary medicine with a focus on respiratory rehabilitation and exercise.

Ongoing arthritis disease activity on abatacept warranted a transition to sarilumab, on which the patient remains stable. Pulmonary management has largely been supportive, and stability has been demonstrated on repeat pulmonary function tests.

## 3. Discussion

We present one of only a limited number of reported cases of COPA syndrome, and in illustrating the retrospective recognition of an archetypal case of COPA syndrome, and we hope to increase awareness of the syndrome among clinicians. Initially diagnosed with RF and anti-CCP seropositive polyarticular JIA in childhood, the patient's early childhood complaints of fatiguability and pneumonia-like episodes were long felt to be unrelated. Upon transition to adult rheumatology care, a thorough review of the patient's history prompted imaging which revealed interstitial lung disease. The patient's cystic lung disease with ground-glass opacities on CT, restrictive spirometry, and clinical course have been classic for COPA syndrome [1–10], though particularly noteworthy is the presence of dual-seropositive, erosive rheumatoid arthritis. The present case illustrates the importance of considering COPA syndrome in patients with JIA and rheumatoid arthritis presenting with extraarticular manifestations and in pediatric patients with respiratory complaints presenting with arthritis. Although rare, multidisciplinary evaluation for COPA syndrome by rheumatology, pulmonary medicine, and genetics might be considered in patients in whom JIA is accompanied by early-onset pulmonary features, regardless of seropositivity, particularly where radiographic and pulmonary function evaluations suggest ILD. Further consideration for extraarticular manifestations should be considered in patients with chronic JIA.

## Data Availability

The data used to support the findings of this study are included within the article.
